# General practitioner views on the determinants of test ordering: a theory-based qualitative approach to the development of an intervention to improve immunoglobulin requests in primary care

**DOI:** 10.1186/s13012-016-0465-8

**Published:** 2016-07-19

**Authors:** S. L. Cadogan, S. M. McHugh, C. P. Bradley, J. P. Browne, M. R. Cahill

**Affiliations:** 1 Department of Epidemiology and Public Health, University College Cork, Cork, Ireland; 2 Department of General Practice, University College Cork, Cork, Ireland; 3 Department of Haematology, Cork University Hospital, Cork, Ireland

**Keywords:** Laboratory testing, Primary care, Interventions, Theoretical domains framework, Behaviour change techniques, Behaviour change wheel

## Abstract

**Background:**

Research suggests that variation in laboratory requesting patterns may indicate unnecessary test use. Requesting patterns for serum immunoglobulins vary significantly between general practitioners (GPs). This study aims to explore GP’s views on testing to identify the determinants of behaviour and recommend feasible intervention strategies for improving immunoglobulin test use in primary care.

**Methods:**

Qualitative semi-structured interviews were conducted with GPs requesting laboratory tests at Cork University Hospital or University Hospital Kerry in the South of Ireland. GPs were identified using a Health Service Executive laboratory list of GPs in the Cork-Kerry region. A random sample of GPs (stratified by GP requesting patterns) was generated from this list. GPs were purposively sampled based on the criteria of location (urban/rural); length of time qualified; and practice size (single-handed/group). Interviews were carried out between December 2014 and February 2015. Interviews were transcribed verbatim using NVivo 10 software and analysed using the framework analysis method. Emerging themes were mapped to the theoretical domains framework (TDF), which outlines 12 domains that can enable or inhibit behaviour change. The behaviour change wheel and behaviour change technique (BCT) taxonomy were then used to identify potential intervention strategies.

**Results:**

Sixteen GPs were interviewed (ten males and six females). Findings suggest that intervention strategies should specifically target the key barriers to effective test ordering, while considering the context of primary care practice. Seven domains from the TDF were perceived to influence immunoglobulin test ordering behaviours and were identified as ‘mechanisms for change’ (knowledge, environmental context and resources, social/professional role and identity, beliefs about capabilities, beliefs about consequences, memory, attention and decision-making processes and behavioural regulation). Using these TDF domains, seven BCTs emerged as feasible ‘intervention content’ for targeting GPs’ ordering behaviour. These included instructions on how to effectively request the test (how to perform behaviour), information on GPs’ use of the test (feedback on behaviour), information about patient consequences resulting from not doing the test (information about health consequences), laboratory/consultant-based advice/education (credible source), altering the test ordering form (restructuring the physical environment), providing guidelines (prompts/cues) and adding interpretive comments to the results (adding objects to the environment). These BCTs aligned to four intervention functions: education, persuasion, environmental restructuring and enablement.

**Conclusions:**

This study has effectively applied behaviour change theory to identify feasible strategies for improving immunoglobulin test use in primary care using the TDF, ‘behaviour change wheel’ and BCT taxonomy. The identified BCTs will form the basis of a theory-based intervention to improve the use of immunoglobulin tests among GPs. Future research will involve the development and evaluation of this intervention.

**Electronic supplementary material:**

The online version of this article (doi:10.1186/s13012-016-0465-8) contains supplementary material, which is available to authorized users.

## Background

Laboratory testing plays an increasingly important role in the diagnosis and monitoring of conditions managed by general practitioners (GPs). An estimated 30 % of all patient encounters result in a test order, and care planning has become increasingly dependent on the results of laboratory tests [1, 2]. This has led to greater scrutiny of the appropriateness of test ordering, with suggestions that as many as 70 % of all tests may be unnecessary depending on the context of care [3–5]. Considerable variation in test ordering patterns by GPs has been identified, further supporting the likelihood that some ordered tests are unnecessary [6–8]. Further, in a recent US survey of over 1700 participants, GPs reported uncertainty about ordering tests in 14.7 % of diagnostic encounters and uncertainty in interpreting results in 8.3 % of these encounters [9]. Healthcare services worldwide are under pressure to reduce their costs, and a review commissioned by the UK Department of Health estimated that costs could be reduced by as much as 20 % by improving utilisation of pathology services [4].

Inappropriate laboratory testing includes both over- and under-utilisation. Overutilisation is wasteful and can increase the likelihood of false positives, poor treatment decisions and adverse outcomes due to unnecessary interventions [5]. Underutilisation may result in morbidity resulting from delayed or missed diagnoses. Overuse and underuse of tests can both lead to longer hospital stays and contribute to legal liability. One large review of laboratory testing patterns found inappropriate testing which was three times higher for low-volume than high-volume tests (32 vs 10 %) [5]. ‘Low volume’ in this study implied a test that was ordered at least ten times less frequently than the most commonly ordered tests [5]. Inappropriate testing is more likely to occur with low-volume tests which may be due to a lack of familiarity with the best treatment practices for the conditions under scrutiny [5].

Our study explores the use of two related low-volume blood tests in primary care, serum immunoglobulin quantitation and immunoglobulin electrophoresis. These tests should be ordered as part of the primary screen for suspected plasma cell dyscrasias (myeloma, lymphoma, chronic lymphatic leukaemia, heavy chain disease and amyloidosis). Immunoglobulins alone may also be requested as part of the diagnostic investigation of patients with recurrent documented infections [10].

Low serum immunoglobulin levels indicate a deficiency of the humoral immune system, while high immunoglobulin levels (with normal electrophoresis) are observed in liver diseases, infections and chronic inflammatory diseases. Raised levels with abnormal electrophoresis may indicate blood dyscrasia [11]. High levels of immunoglobulins are a feature of many clinical conditions in older patients but are only really diagnostically useful in specific haematological disorders such as myeloma and lymphoma. The clinical features of these conditions can be vague and non-specific and overlap with the symptoms of a wide range of other conditions. Thus, in older patients, immunoglobulin testing is probably best undertaken as a second-line investigation where there are other tests (such as a full blood count) which indicate the possibility of a blood dyscrasia. Knowing when to order immunoglobulins, therefore, can be challenging for GPs and may require a clinical judgement in the context of rather non-specific clinical features. Their interpretation is also difficult and often requires specialist input. Furthermore, once abnormal levels have been detected, this can lead to other costly activities such as referral to secondary care that may ultimately prove to have been futile. To date, no previous studies have studied GPs’ serum immunoglobulin test ordering behaviour.

A recent systematic review identified a number of effective interventions for reducing inappropriate test ordering, defined as testing practices that do not lead to patient benefits [12]. Educational strategies [13–15], cost displays [16], changing order forms [17] and various methods of disseminating guidelines [18, 19] displayed positive effects. Ten out of 11 studies included in the review found significant reductions in the volume of tests following an intervention, with effect sizes ranging from 1.2 to 60 % [12]. However, the positive effects of these interventions were often short term and none lasted longer than 2 years [12]. Implementation science experts have suggested that theory-based targeted behaviour change techniques may maximise the potential long-term effects of such interventions [20]. In particular, there is a need to identify the key enablers and barriers to successful implementation of interventions in this area and to improve their design so that sustainability is ensured [21].

**Fig. 1:**
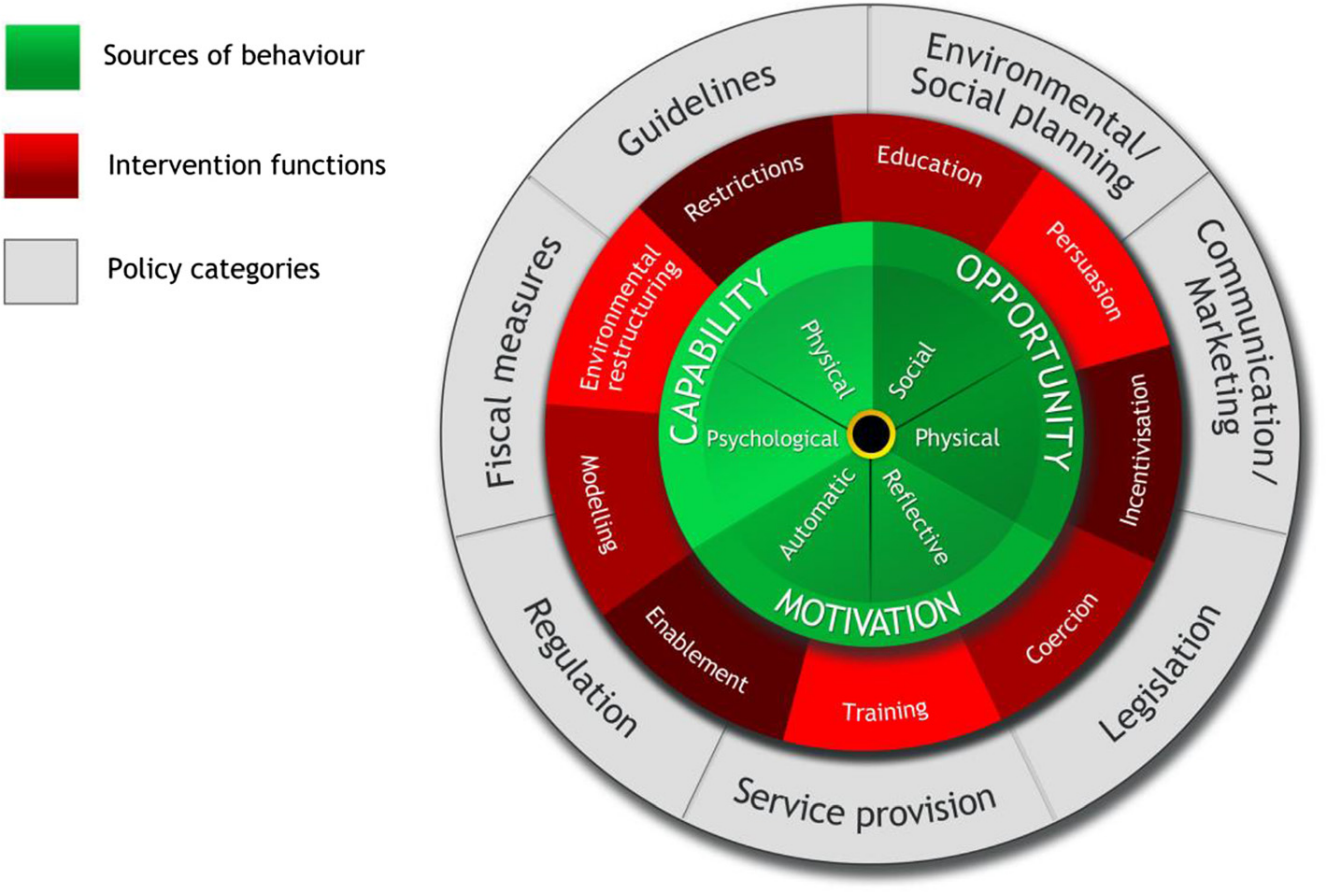
The behaviour change wheel. Reproduced with permission from Michie et al. [30]

A growing body of literature supports the use of psychological theories in the development of behaviour change interventions [20, 22]. In particular, recent guidelines emphasise the need to report three aspects of behaviour change interventions [23]: the use of psychological theory to identify the factors which influence the target behaviour change (i.e., ‘mechanism of action’); the ‘active ingredients’ of behaviour change interventions (i.e., the intervention content) and how this was delivered (i.e., who the intervention targeted, who delivered it and in what format and setting). The theoretical domains framework (TDF) has been identified as a useful tool for identifying the ‘mechanism of action’ and selecting behaviour change techniques (BCTs) to include in behavioural change interventions [24, 25]. The TDF is an elaboration of the six capability, opportunity and motivation conditions of the ‘behaviour change wheel’ known as the COM-B model [26] (see Fig. 1). To date, a number of empirical studies have used the TDF to explore the implementation of BCTs with GPs including low back pain management [20] and medication prescribing [27].

The aims of this study were to use the TDF and corresponding COM-B to identify the enablers and barriers to altering immunoglobulin test ordering behaviour from the perspective of GPs, and to use this information to identify the corresponding BCTs and feasible intervention strategies to align requesting practice with possible health gain, for future evaluative research. The specific GP behaviours targeted in this study were serum immunoglobulin test requests that were not correctly aligned with the presenting symptoms of patients or were unlikely to lead to patient benefit.

## Methods

### Study design

Qualitative semi-structured interviews were conducted with GPs in two adjacent counties in the Republic of Ireland.

### Sampling and recruitment

All 587 practising GPs in the Cork-Kerry region were identified using a list provided by the Health Service Executive, the organisation that provides public healthcare in the Republic of Ireland. GPs were stratified by the volume of tests they had requested over the previous two years from the primary public hospitals in the region that provide the relevant laboratory services (Cork University Hospital and University Hospital Kerry). This involved categorising GPs into low (<10 tests/year), moderate (10–50 tests/year) and high (>50 tests/year) requesters for immunoglobulin tests. Within each category of requesting patterns, GPs were then purposively sampled based on the sampling criteria of location (urban/rural); length of time qualified (<10 years, 10–20 years, >20 years) and practice size (single-handed/group). GPs were sent a written invitation letter and study information sheet, followed by a telephone call to determine if they were interested in participating. A′10 plus 3′ method, which has been previously recommended for theory-based interview studies, was used to determine our initial sample size target [28]. Ten GPs were interviewed, and the material collected was analysed at this point. Three further GPs were then interviewed to check if any new insights were produced. If further interviews were deemed necessary, they would be conducted in blocks of three with a check for data saturation at the end of each block. One additional block of three interviews was necessary to reach data saturation, giving a total of 16 interviews. Written informed consent was obtained from all participants prior to the interview.

### Semi-structured interview process

Face-to-face semi-structured interviews were carried out in the primary care setting by one researcher (SLC) between December 2014 and February 2015. The interview topic guide was developed based on the second version of the TDF [29] and discussion among the authors and is summarised in Additional file 1: Table S1. The topic guide and interview process were piloted by interviewing two GPs. Following this pilot, there were no changes to the topic guide, but refinements were made to the probes that were used to explore GP responses and to the interviewing style. These pilot interviews were facilitated in the same manner as the remaining interviews and are included in the final analysis with consent from the interviewees. Interviews were recorded and transcribed verbatim. NVivo 10 software was used to facilitate data analysis.

### Data analysis

#### Stage 1

Data analysis followed the framework analysis approach [30]. Data familiarisation was carried out by rereading the transcripts and listening back to interview recordings. Following open-coding, emergent themes were mapped onto the domains of the TDF and corresponding capability, opportunity and motivation conditions. When themes were relevant to more than one domain, they were initially coded to both domains. All transcripts were coded by the researcher who conducted the interviews (SLC), and a subset of six interviews (including a sample of urban/rural, male/female and more/less experienced GPs) were independently coded and analysed by a second researcher (SMMH) as a method of verification of the initial analysis. Coding and mapping by the independent researchers (SLC and SMMH) were compared. There were no major disagreements. Minor differences arose in relation to the mapping of codes to TDF, particularly when codes mapped to more than one domain. Any differences were resolved by consensus discussion; the researchers referred back to the original transcripts to reassess the context of the codes and discussed the particular code in light of the breadth of data from other transcripts mapped to that TDF drawing on SLC’s knowledge of all the interviews conducted and analysed.

#### Stage 2

The BCT taxonomy, version 1, was then used to recommend potential ‘intervention components’, that is, strategies to improve laboratory testing in primary care [24, 31]. This taxonomy has been developed in order to standardise the content and reporting of intervention studies [24] and includes 93 BCTs grouped within 16 categories with detailed definitions of each [24]. This process involved mapping the BCTs to the TDF and corresponding COM-B identified in stage 1 [31]. The full list of BCTs has previously been applied to the TDF by a group of behaviour change experts [25]. We used this list [25] along with a more recently published list (resulting from an expert mapping exercise) [32] as a reference tool for guiding our selection of BCTs. Our multidisciplinary research team reviewed the BCTs that had been mapped to key domains in order to reach consensus on the BCTs that should be selected for the intervention development. This selection process was guided by the interview data and focused on identifying barriers and facilitators that could feasibly be targeted based on the available intervention resources. For example, if time and perceived workload were reported as major barriers to GP testing behaviours, BCTs that could be delivered more efficiently were to be prioritised.

## Results

### Sample demographics

In total, sixteen GPs were interviewed, including ten males and six females. Table 1 provides details of the participants’ characteristics categorised by location type (urban/rural).

**Table 1:** Characteristics of GPs interviewed based on urban/rural location

GP characteristics	Urban (*n* = 8)	Rural (*n* = 8)	Total (*n* = 16)
Gender			
Male	5	4	9
Female	3	4	7
Age group			
30–40	2	2	4
40–50	4	2	6
50–60	0	2	2
>60	2	2	4
Training practice^a^			
Yes	5	5	10
No	3	3	6
Practice nurse^b^			
Yes	5	5	10
No	3	3	6
Practice type			
Solo GP	2	2	4
Group practice	6	6	12

^a^Training practice: a practice that facilitates trainee GPs on the Irish medical training scheme

^b^Practice nurse refers to whether the practice has a practice nurse employed

### Summary of findings from analysis at the level of theoretical domains

The analysis identified seven domains of the TDF that were relevant to 18 emerging themes (Table 2). These findings are described in greater detail below. The remaining domains that were not identified (intention, optimism, goals, emotion, social influences) are not discussed as not enough references to the relevant constructs were made.

**Table 2:** TDF identified and the corresponding key themes that evolved

TDF	Themes
Knowledge^a^	▪ Limited knowledge of when to use immunoglobulin tests effectively. ▪ GPs expressed difficulty with interpreting the results (particularly borderline abnormal results). ▪ Lack of knowledge of how to effectively manage patients when the result is abnormal (when to refer).
Environmental context and resources	▪ Lack of clear guidelines on when to use an immunoglobulin test in primary care. ▪ Need for instructions on when to refer patients with abnormal results.
Beliefs about consequences	▪ Excessive follow-up workload that comes with doing an immunoglobulin test.
Beliefs about capabilities	▪ Feel they are poor at triaging patients for potential myeloma. ▪ GPs find interpreting immunoglobulin results difficult. ▪ Concern at what to do with an abnormal test result, in particular, borderline abnormal results.
Social/professional role	▪ Happy to do the tests for specialist monitoring purposes. ▪ Many GPs feel it is not a common test in primary care.
Memory, attention and decision process	▪ A follow-up test performed on the basis of results of another test. ▪ Not a priority in primary care. ▪ Older patients with chronic back pain trigger the test for many. ▪ Small minority use them regularly for screening.
Behavioural regulation	▪ Education and guidelines mentioned the most. ▪ Electronic strategy highlighted as feasible/system-level strategy. ▪ Multidisciplinary approach.

^a^Knowledge and skills were merged due to overlapping constructs

The main domains from the TDF which emerged were ‘knowledge’, ‘skill’, ‘environmental context and resources’, ‘social/professional role and identity’, ‘beliefs about capabilities’, ‘beliefs about consequences’, ‘memory, attention and decision making processes’ and ‘behavioural regulation’.

#### Knowledge and skill

The domains knowledge and skill were merged as the constructs overlapped. That is, GPs primarily referred to ‘procedural’ knowledge, and where competence or ability (skills) was mentioned, it was always linked to a lack of knowledge. Participants reported that knowledge was a key barrier when requesting immunoglobulins. In particular, GPs identified a number of different scenarios when they would request the test including ‘recurrent infections’, ‘respiratory problems’ and ‘back pain’. The majority of GPs stated that they would be considering potential ‘myeloma’ when they are requesting; however, a small minority of GPs identified other conditions including ‘rheumatoid arthritis’ and ‘anaemia’. GPs reported that a need for greater guidance and training on when to use serum immunoglobulin tests would be beneficial.

“*No*,* my natural* *feeling towards it would be I feel that I don't know enough about them. And I might be getting more value out of them if I knew more you know yeah*”. (GP 6)

“*It’s confusing I mean it’s just a confusing area for us and when do we request them anyway?*” (GP 2)

The interpretation of immunoglobulin results was identified as a challenge by almost all GPs interviewed. In particular, they discussed difficulty interpreting borderline abnormal results and making treatment decisions for these cases.

“*Well it can be difficult to interpret. So often if there is a difficulty with them you basically have to ring the lab to confirm before you discuss with the patient because at that stage anyway you're talking about referring the patient on anyway*”. (GP 3)

“*I find them very hard to interpret and I end up ringing haematology about the interpretation*”. (GP 4)

#### Environmental context and resources

The majority of GPs mentioned a lack of clear guidelines on when to request an immunoglobulin test. GPs reported that they often phone the laboratory for advice in the absence of clear requesting and interpretative guidelines. GPs highlighted that this was time-consuming; however, they commended the support of laboratory staff.

“*I find the labs very good to be honest with you. They'll put you onto the consultant that's on duty at the time or if not they'll ring you back. So I've never had a problem with it*”. (GP 09)

Patient management post testing was also mentioned as a concern, in particular a lack of information on the usefulness of the test for managing patients in primary care. For example, one GP used the test for screening patients but expressed that he did not know what to do with the result.

“*…I suppose there are some issues like that I have to get advice from the haematologists where I'm you know at a loss where do we go from here. Do we just put it up on their record and leave it there as background information or do we proceed further with investigations*”. (GP 10)

#### Beliefs about capabilities

In general, the majority of GPs admitted a lack of confidence in their ability to use serum immunoglobulin tests, in particular managing their patients who receive an abnormal result. Many reported that they often had to phone the laboratory and speak to the haematology registrar or consultant. Other stated that they often just referred these patients.

“*I feel my ability to triage them is poor, and you don't want to be ringing the haem reg (haematology registrar) all of the time*”. (GP 13)

“*So, if I get an abnormal result, I'd either want to speak to a haem reg (haematology registrar) or refer on because I wouldn't be confident in managing an abnormal result*”. (GP 11)

“*If it's minor and it's fractional and the patient is well, I'm happy to do nothing. If it’s significant and I'm just unsure that's when I ring the reg (haematology registrar) or consultant*”. (GP 4)

#### Beliefs about consequences

GPs stated that the workload created by doing the tests was a deterrent, in particular having to liaise with the laboratory or haematology department.

“*Exactly, because ironically in my experience doing immunoglobulins will lead to workload perhaps because I'm going to have to liaise with my hospital specialist colleagues ah because to get their opinion on them actually*”*. (GP15)*

Also, a small number of GPs mentioned ‘fear of litigation’ and ’fear of missing a myeloma’ as other potential consequences leading to what they described as potentially ineffective use of the test. Again, these consequences related back to their perceived lack of knowledge.

#### Social/professional role and identity

Some GPs reported that they request a large volume of immunoglobulins, often as a screening test. However, the majority of GPs referred to the test as a second- or third-line test, performed subsequent to previous tests. They stated that they considered immunoglobulins a rare test and one they would not perform regularly.

“*We have a reg (registrar) and am we have two practice nurses and at the induction for the registrar there are certain blood tests we advise them not to perform regularly, this would be one of the ones we advise not to perform regularly*”. (GP 4)

GPs highlighted that while they feel the test should be available in primary care, they often consider it a secondary care test and in few cases highlighted their gatekeeping role in using the test.

“*You see GP's often wouldn't see them because if they are being monitored by haem (haematology). In fairness GP practices do them, but they probably do some with their forms to their nurse but the GP won't see them. And often, if they come in with the form, it is the consultant’s name of the form and their results don't come back to us*”. (GP 13)

Finally, younger female GPs (in their 30s) suggested that they may request fewer tests due to their patient demographics. They commented that the majority of their patients are younger females and children who are less likely to require the test.

“*I see pretty much all women and children and so I would have very little need. There is a very small number of older patients*”. (GP 13)

“*I think that because I am a young female GP I don’t see a huge pile (volume) of older patients – so what I do is kiddies (children) and contraception and a lot of antenatal care and gynae (gynaecological) stuff*”. (GP 09)

In particular, each of them highlighted that patients often grow with them (the GP) in terms of age and suggested that more experienced male GPs may appropriately request more.

#### Memory, attention and decision process

When asked about what prompts them to perform the test, GPs had many contrasting reasons, with many highlighting that they were unsure of when to do them. GPs discussed some key symptoms that would influence their decision-making process such a ’chronic back pain’, ‘persistent infections’ or in some cases simply ‘age’. These factors varied between GPs; however, almost all GPs mentioned myeloma as the potential endpoint diagnosis from doing the test.

“*I use them primarily in situations where I think there might be something significant. So usually you tend to see it used in say recurrent infections or more particularly in patients who might potentially have multiple myeloma*”. (GP 5)

Some GPs mentioned that they request the test to monitor patients with an existing diagnosis at the request of the consultant, where results of previous tests are available.

“*Once they have been into the consultant and the consultant has said this is…just check it every 12 months, that's fine by me. I'll check it if its. I'll look at the results. I can very easily compare them to the last results on my computer and I can tell if it’s changing or if it's not changing*”. (GP 2)

#### Behavioural regulation

GPs suggested potential strategies to help improve testing in primary care. GPs primarily requested education and guidelines on when to test and also on how to interpret the results.

“*I guess education is what we need really. You know what value it will be to us for our patient and for, obviously for information for helping the patient*”*. (GP 4)*

“*Yeah I suppose we need a one page protocol so we know where we are going with this thing you know*”. (GP 10)

In particular, they discussed the feasibility and receptiveness of educational-based interventions in primary care. A key barrier according to the GPs was ensuring sustainable strategies are selected.

“*Well I suppose the easy answer would be to say training or education or a booklet or a pamphlet but there's a great risk that if you produce a document like that it will be quickly glanced at, thrown in the bin, or what I would be doing, I would put it in a filling place and I'd never look at it again. So you need to do something that is sustained and continuous actually I would say*”. (GP 4)

“*I think the education strategy has to be built in with reminders*”. (GP 2)

Two GPs discussed penalties, incentives, feedback and restrictive strategies. However, when discussed in detail, GPs concluded that the lack of knowledge would potentially hamper the effects of such strategies.

“*Another thing of course would be either penalty or incentivisation whereby if and it's a sad thing to say but we respond to incentives. So if you over-prescribe, sorry over-request to a ridiculous degree you know four times the average then there should be some kind of a penalty. Or, if you're within certain ranges some kind of incentive. And, I think that would affect real change*”. (GP 12)

“*I think am the form…if you want a particular test that is out of the ordinary, I think that you should have to justify your reasons in the clinical details box for the test*”. (GP 09)

Importantly, GPs stressed that a system-level approach needs to be followed, where possible at the laboratory level providing education or the use of an algorithm.

“*I think it has to be at systems level. Cause (because) I think a once off workshop or once off piece of paper coming out to the practice won’t make a difference here. It has to be at systems level. I think it has to be centrally delivered from the Department of Haematology*”. (GP 14)

“*Well I suppose if you had your algorithm so if you had like so complaints or five scenarios where so if the come in with this you should be ordering an S pep and you should be looking for such and such on the results. So if you did it that way*”. (GP 16)

“*Yeah, maybe like a performer or an algorithm or some kind may be useful alright*”. (GP 12)

However, GPs mentioned that any strategy developed to improve the use of laboratory tests should be primary care responsive and consider the differing motivations of GPs versus specialist physicians. For example, many GPs stated that they perform laboratory tests to ‘rule out’ a diagnosis while specialist physicians may be more likely to carry out tests to ‘rule in’ or confirm a diagnosis.

“*You know because a lot of them, if they come back abnormal are very useful you know and this is one of the difference between general practice and hospital practice, the importance of normal blood tests. General practitioners generally do blood tests to out rule illnesses and hopefully getting normal results, whereas hospital practice would be much more inclined to do a blood test to confirm an abnormal result or to look for an abnormal result*”. (GP 06)

#### Application of BCT taxonomy and identification of potential intervention functions

Table 2 shows the final mapping of the BCTs to the identified TDF and COM-B components. Using previous work on mapping BCTs to the TDF, as outlined in the methods, the research team identified and selected seven BCTs with potential for inclusion in a future intervention involving GPs. This resulted in six of the 16 BCT groupings: ‘shaping knowledge’, ‘feedback and monitoring’, ‘natural consequences’, ‘comparison of outcomes’, ‘antecedents’ and ‘associations’. Within these six groupings, seven specific BCTs were found to be relevant (definitions of each of these can be found in Additional file 2: Table S2). For example, the technique ‘instructions on how to perform the behaviour’ from the 93-item BCT taxonomy [24] was selected to target the GPs’ lack of knowledge on when to request the test, while another technique ‘prompts/cues’ was used to target the feasibility of implementing an education-based strategy. A full description of the selection and exclusion of BCTs can be found in Additional file 3.

Using these BCTs, four of the ten intervention functions were deemed potentially useful for developing an intervention targeting the GP population. Selected BCT functions included ‘education’, persuasion’, ‘environmental restructure’ and enablement’. Subsequently, potential intervention components were devised and include the following: providing guidelines on when to request the test, clearly communicating situations where testing is not beneficial for patient care education and giving advice (attached to results) on how to interpret results and manage patients with abnormal levels. These intervention strategies were also suggested by GPs during the interviews and are likely to assist in the development of feasible and welcome interventions. Table 3 provides details of the mapping process for selecting the BCTs and intervention components. For example, for the domain knowledge, the BCTs’ ‘information on how to perform the behaviour’ and ‘feedback on behaviour’ were selected. However, providing feedback on individual GP requesting patterns was deemed potentially inappropriate due to the lack of knowledge GPs expressed around when to request the test in the first instance.

**Table 3:** Suggested intervention content and mechanisms of action using the behaviour change technique (BCT) taxonomy (v1); behaviour change wheel capability, opportunity and motivation-behaviour (COM-B) model; and theoretical domains framework (TDF) [26]

Intervention component	Mechanisms of action		Intervention content		
	TDF^1^	COM-B^2^	BCT grouping	BCTs	Functions
Information and training about immunoglobulin use in primary care, i.e. provide guidelines on when to request and how to interpret results	Kn, MAD, BR	C-(Psy.), C-(Phys.)	Shaping knowledge	Instructions on how to perform behaviour	Education
Provide feedback on individual GP feedback (volume of tests) Note: not a suitable strategy in this context	Kn,	C-(Psy.)	Feedback and monitoring	Feedback on behaviour	Education, persuasion
Clearly communicate situations where immunoglobulin testing is not beneficial. (i.e. develop an algorithm of scenarios where tests should be performed, supported by consultant haematologists and GPs)	B Cap, B Con	M-(Refl.)	Natural consequences, comparison of outcomes	Information about health consequences, credible source	Persuasion
Provide notes detailing consultant advice on the test results (ideally provided on the end of the test results)	Env, S/P Id	O-(Phys.), O-(Soc.)	Antecedents, associations	Restructuring the physical environment, prompts/cues, Adding objects to the environment	Environmental restructure, enablement

^1^TDF abbreviations: *Kn* knowledge, *MAD* memory, attention and decision processes, *BR* behavioural regulation, *Env* environmental context and resources, *B Cap* beliefs about capabilities, *B Con* beliefs about consequences, *S/P Id* social/professional role and identity

^2^COM-B components: *C-(Psych)* psychological capability, *C-(Phys)* physical capability, *M-(Refl)* reflective motivation, *O-(Phys)* physical opportunity, *M-(Auto)* automatic motivation

The final mapping of the relevant BCTs and corresponding intervention components to the COM-B and TDF models can be found in Table 4.

**Table 4:** Final mapping of BCTs relevant to design a strategy for improving immunoglobulin test use in primary care

				Capability			Opportunity		Motivation	
				Psychological			Physical	Social	Reflective	
BCT group [24]	BCT [38]	Functions	Support from interviews	Kn*	MAD	BR	Env	S/P Id	B Con	B Cap
Feedback and monitoring	Feedback on behaviour	Education	GPs reported that they are not aware of how many tests they request, or if they request tests appropriately.	✓						
Shaping knowledge	Instructions on how to perform the behaviour	Education	GPs expressed lack of knowledge about when to do the test and asked for standardised guidance or resources.	✓	✓	✓				
Associations	Prompts and cues	Enablement	GPs highlighted that a once off education strategy is not desirable. Instead, they suggested a reminder on test results.					✓		
Comparison of outcome	Credible source	Persuasion	GPs mentioned the importance of input from specialists with regard to patient management following an abnormal test result.						✓	
Antecedents	Restructuring the physical environment	Environmental restructure	GPs discussed current requesting procedure as a potential target (requesting more detail on order forms.				✓			
Antecedents	Adding objects to the environment	Environmental restructure	GPs discussed lack of guidelines for interpreting test results and expressed interest interpretive comments on test results.				✓			
Natural consequences	Information about health consequences	Persuasion	GPs expressed concern over the consequences of not performing a test in terms of missing a myeloma diagnosis.							✓

* TDF domain abbreviations: Kn knowledge, MAD memory, attention and decision processes; BR behavioural regulation; Env environmental context and resources; B Cap, beliefs about capabilities; B Con, beliefs about consequences; S/P Id, social/professional role and identity

## Discussion

This study presents a systematic, theory-based approach to developing an intervention to improve test ordering in primary care. We found that serum immunoglobulin test ordering is influenced by many social and contextual factors. Using the BCT taxonomy, TDF and COM-B models, four potentially useful intervention functions on which to model future interventions have been identified. These are education, environmental restructuring, enablement and persuasion by specialists.

Evidence suggests that medical professionals respond differently than other healthcare professionals to interventions designed to change their behaviour [33] and that interventions are more likely to influence change if they target the factors underlying barriers to behaviour change [25]. The barriers to behaviour change also differ across healthcare professionals and may result from differences in training, knowledge, work experience, personality and other individual characteristics [34]. To our knowledge, this is the first study to identify these barriers in the primary care setting and develop potential strategies for improving test ordering using clearly delineated behaviour change theories. Two key barriers identified were lack of knowledge on when to use immunoglobulin tests in primary care and how to interpret the results. Until these knowledge deficits are addressed, an audit and feedback approach to behaviour may be unsuccessful as the underlying drivers of inappropriate test ordering will not have been addressed. This is in line with the rejection of audit and feedback by the interviewees and the findings of previous studies [35, 36].

Interviewees emphasised that the passive provision of information alone is not sufficient to bring about behaviour change in primary care. The GPs interviewed argued that highlighting a discrepancy between expected and actual test ordering rates may be perceived as judgmental of their professional capacity. In particular, they discussed their clinical motivation for performing tests and the context of primary care setting as key characteristics that should be considered [37].

Interviewees suggested that helping them to improve their knowledge through, for example, educational reminders or external support was likely to be successful. This support should come from specialists (‘a credible source’) and incorporate the dissemination of guidelines and feedback on how best to manage the patient. This is consistent with other studies on the value of education-based specialist support strategies such as interactive educational sessions coupled with the use of local opinion leaders/or feedback reports/or feedback reports [12, 15, 18]. The interviewed GPs also suggested that strategies should be designed at the laboratory level, such as changing the order forms or adding interpretive guidance to the results.

When designing specialist support services to guide test ordering, strategies should be responsive to the needs of both GPs and laboratory services. For example, while GP knowledge may be a barrier to behaviour change, strategies aimed at targeting testing behaviour also need to consider the motivation for testing in primary care, which may be to rule out a diagnosis rather than to rule in one. This may require educational messages to draw on a different knowledge base, for example, regarding the negative predictive value of the test rather than just information about the positive predictive value.

Our research suggests that specialist support should be provided from a credible source in an encouraging and non-judgemental manner. In the instance of immunoglobulins, haematologists are the best equipped to do so and therefore are well placed to assist the laboratory to formulate advice and comment on test interpretation. This support may best be provided in the form of interactive learning sessions with local opinion leaders and feedback reports generated at the laboratory level. Basic medical education could also incorporate information on immunoglobulin testing guidelines and interpretation.

### Strengths and limitations

A key strength of this study is the systematic approach followed to identify key theoretical domains and select BCTs to support the development of an intervention in primary care. In doing so, we have followed existing recommendations on designing theory-informed behaviour change interventions [20]. By making the relationship between the supporting theoretical framework and our intervention development explicit, it may be easier to identify how different elements of any subsequently designed intervention contribute to observed behaviour change. Also, in addition to identifying mediators of behaviour change to target using an intervention, the interviews have supplied valuable information about the clinical context in which the behaviours are currently performed. This information, along with the findings of our previous review [12], will inform decision-making around which intervention approaches should be employed in future research by our research group.

There are some limitations to this study. First, the findings reflect GPs’ perceptions of influences on their clinical behaviours, but we do not have data on their actual behaviour in specific cases. Finally, we have drawn on a particular set of psychological theories of behaviour change, but there may be alternative theories or frameworks that might also be applicable to explain test ordering behaviour of primary care physicians.

## Conclusions

This research provides an important overview of the behavioural factors influencing laboratory testing among GPs. The incorporation of behavioural theory, specifically the COM-B, TDF and BCT taxonomy, has supported the identification of factors such as knowledge and the social and environmental context, which are key for understanding testing behaviours. Selected BCTs provide the groundwork for developing a theory-based intervention to improve appropriate immunoglobulin testing in primary care. Future work will involve developing and evaluating an intervention using the selected BCTs.

## Abbreviations

BCT, behaviour change technique; TDF, theoretical domains framework; COM-B, capability, opportunity and motivation-behaviour; GP, general practitioner

## Additional files

Additional file 1: Table S1. Topic Guide using TDF framework. (DOCX 19 kb)

Additional file 2: Table S2. BCT groups and corresponding BCTs and definitions. (DOCX 16 kb)

Additional file 3: Mapping of behaviour change techniques (BCTs) to key domains for inclusion in an intervention targeting immunoglobulin testing behaviour of general practitioners. (DOCX 20 kb)
