# Supplementary material for: General practitioner views on the determinants of test ordering: a theory-based qualitative approach to the development of an intervention to improve immunoglobulin requests in primary care
Source: Implement Sci. 2016 Jul 19;11:102. doi: 10.1186/s13012-016-0465-8 (PMC4952272; doi:10.1186/s13012-016-0465-8)
Supplement: Supplementary file 1 — Topic Guide using TDF framework. (DOCX 19 kb) [file 13012_2016_465_MOESM1_ESM.docx]

**Additional file 1** – Table S1: Topic Guide using TDF framework

| TDF Domains | TDF Definitions (Constructs)* | Prompts/questions |
| --- | --- | --- |
| Knowledge | An awareness of the existence of something. (Knowledge including knowledge of condition/scientific rationale. Procedural knowledge. Knowledge of task environment.) | - Are you familiar with any guidelines for requesting immunoglobulins? - Are you comfortable interpreting results of immunoglobulin tests? - Knowledge their own and other GP requesting patterns (procedure knowledge) - Knowledge about problems associated with requesting (knowledge of task environment) |
| Skills | An ability or proficiency acquired through practice. (Skills Skills development Competence Ability Interpersonal skills Practice Skill assessment) | - Do you know how to do it/able to use tools? |
| Social professional role and identity | A coherent set of behaviors and displayed personal qualities of an individual in a social or work setting. (Professional identity Professional role Social identity Professional boundaries Professional confidence Group identity Leadership Organizational commitment) | - When would you request an immunoglobulin test? - Do you think immunoglobulins are important/appropriate part of your role? |
| Beliefs about capabilities | Acceptance of the truth, reality, or validity about an ability, talent, or facility that a person can put to constructive use. (Self-confidence Perceived competence Self-efficacy Perceived behavioral control Beliefs Self-esteem Empowerment Professional confidence) | - How confident would do you feel about requesting immunglobulin tests? - Any difficulties in interpreting results or deciding when to test? |
| Beliefs about consequences | Acceptance of the truth, reality, or validity about outcomes of a behaviour in a given situation. (Beliefs Outcome expectancies Characteristics of outcome expectancies Anticipated regret Consequences) | - How do you deal with an abnormal result? |
| Intention | A conscious decision to perform a behavior or resolve to act in a certain way. Mental representations of outcomes or end states that an individual wants to achieve. (Stability of intentions Stages of change model Trans-theoretical model and stages of change Implementation intention) | - Why do you do these tests? |
| Goals | Goals (distal/proximal) Goal priority Goal/target setting Goals (autonomous/controlled) Action planning | - What are your key priorities for requesting this test? - Do you have an action plan/criteria for performing the test? |
| Memory, attention and decision processes | The ability to retain information, focus selectively on aspects of the environment and choose between two or more alternatives. (Memory Attention Attention control Decision making Cognitive overload/tiredness) | - Is it something you do routinely? |
| Environmental context and resources | Any circumstance of a person’s situation or environment that discourages or encourages the development of skills and abilities, independence, social competence, and adaptive behavior. (Environmental stressors Resources/material resources Organizational culture/climate Salient events/critical incidents Person x environment interaction Barriers and facilitators) | - Do resources influence whether you perform the test (e.g. any guidelines you follow)? - Are there clear guidelines available? - Are there clear communication channels (laboratories/ consultants etc)? - Adequate communication telephone/written correspondence with Consultants prior to referral - Useful interaction with consultants where necessary |
| Social influences | Those interpersonal processes that can cause individuals to change their thoughts, feelings, or behaviors. (Social pressure Social norms Group conformity Social comparisons Group norms Social support Power Intergroup conflict Alienation Group identity Modeling) | - Do you seek opinions of colleagues in decision making / interpreting test results? - Why might there be variation in requesting patterns between GPs |
| Emotion | A complex reaction pattern, involving experiential, behavioral, and psychological elements, by which an individual attempts to deal with a personally significant matter or event. (Fear  Anxiety Affect Stress Depression Positive/negative effect Burn-out) | - If appropriate…indirectly probe for any significant reasons such a fear of missing something or fear of litigation as motivators for testing. |
| Behavioral regulation | Anything aimed at managing or changing objectively observed or measured actions. (Self-monitoring Breaking habit Action planning) | - Are there any guidelines you follow? - Probe for potential strategies that may support effective test use. |
| Optimism | The confidence that things will happen for the best or that desired goals will be attained  (Pessimism  Unrealistic optimism  Identity) | - If appropriate... responsiveness of GPs/ feasibility of mentioned strategy (if GP suggests strategy for behavior change) |
| Reinforcement | Increasing the probability of a response by arranging a dependent relationship, or contingency, between the response and a given stimulus  (Incentives  Punishment  Consequents  Reinforcement  Contingencies  Sanctions) | - If appropriate….probe about sustainability of strategies for effective testing behaviour. |

*Definitions and constructs: Cane J, O’Connor D, Michie S: **Validation of the theoretical domains framework for use in behaviour change and implementation research**. *Implement Sci* 2012, **7**(1):37.
