# Supplementary material for: General practitioner views on the determinants of test ordering: a theory-based qualitative approach to the development of an intervention to improve immunoglobulin requests in primary care
Source: Implement Sci. 2016 Jul 19;11:102. doi: 10.1186/s13012-016-0465-8 (PMC4952272; doi:10.1186/s13012-016-0465-8)
Supplement: Supplementary file 2 — BCT groups and corresponding BCTs and definitions. (DOCX 16 kb) [file 13012_2016_465_MOESM2_ESM.docx]

| Additional file 2 – Table S2: BCT groups and corresponding BCTs and definitions | |  |
| --- | --- | --- |
| BCT grouping [[24](#_ENREF_24)] | **Relevant BCT and definition [**[**37**](#_ENREF_37)**]** |  |
| 1) Goals and planning | **N/A** |  |
| 2) Feedback and monitoring | **Feedback on behaviour:**  - Monitor and provide informative or evaluative feedback on performance of the behaviour |  |
| 3) Social support | **N/A** |  |
| 4) Shaping knowledge | I**nstructions on how to perform the behaviour**  - Advise or agree on how to perform the behaviour (includes ‘skills training’) |  |
| 5) Comparison of behaviour | **N/A** |  |
| 6) Associations | P**rompts and cues**  - Introduce and define environmental or social stimulus with the purpose of prompting or cueing the behaviour. The prompt or cue would normally occur at time/place of performance |  |
| 7) Repetition and substitution | **N/A** |  |
| 8) Comparison of outcomes | **Credible source**  - Present verbal or visual communication from a credible source in favour of or against the behaviour |  |
| 9) Reward and threat | **N/A** |  |
| 10) Antecedents | **Restructuring the physical environment**  - Change, or advise to change the physical environment in order to facilitate performance of the wanted behaviour or create barriers to the unwanted behaviour (other than prompts/cues, rewards/punishments)  **Adding objects to the environment**  - Add objects to the environment to facilitate performance of the behaviour |  |
| 11) Identity | **N/A** |  |
| 12) Scheduled consequences | **N/A** |  |
| 13) Self-belief | **N/A** |  |
| 14) Natural consequences | **Information about health consequences**  - Provide information (e.g. written, verbal, visual) about health consequences of performing the behaviour |  |
| 15) Regulation | **N/A** |  |
| 16) Covert learning | **N/A** |  |
