# Supplementary material for: General practitioner views on the determinants of test ordering: a theory-based qualitative approach to the development of an intervention to improve immunoglobulin requests in primary care
Source: Implement Sci. 2016 Jul 19;11:102. doi: 10.1186/s13012-016-0465-8 (PMC4952272; doi:10.1186/s13012-016-0465-8)
Supplement: Supplementary file 3 — Mapping of behaviour change techniques (BCTs) to key domains for inclusion in an intervention targeting immunoglobulin testing behaviour of general practitioners. (DOCX 20 kb) [file 13012_2016_465_MOESM3_ESM.docx]

| **Additional file 3: Mapping of behaviour change techniques (BCTs) to key domains for inclusion in an intervention targeting immunoglobulin testing behaviour of General Practitioners** | | | |
| --- | --- | --- | --- |
| **TDF Domain** | **BCTs identified using Cane et al [32]*** | **BCTs identified using Michie et al [24]*** | **Selected and excluded BCTS** |
| **Knowledge** | 1. Feedback on behaviour  2. Biofeedback  *3. Antecedents*  *4. Health consequences* | *5. Instructions on how to perform behaviour* | Selected BCT: 3, 4, 5  BCT 3/5: Provide information and training about immunoglobulin use in primary care, i.e. provide guidelines on when to request and how to interpret results.  BCT 4: Clearly communicate situations where immunoglobulin testing is not beneficial. (i.e. develop an algorithm of scenarios where tests should be performed)  Non-selected BCTs: 1, 2  BCT 1: Individual GP feedback on requesting patterns not within the scope of this project  BCT 2: Not relevant for the context of the study |
| **Memory, attn. & decision making** | none | 1. Self-monitoring  2. Planning, implementation  *3. Prompts, triggers, cues* | Selected BCTs: 3  Mapped to behaviour regulation – see below for description)  Non-selected BCTs: 1,2  BCT 1: Not feasible for GPs to monitor behaviour in this instance.  BCT 2: not applicable for interventions in this setting. |
| **Environmental context & resources** | *1. Restructuring the physical environment*  2. Restructuring the social environment  3. Avoidance/changing exposure to cues  for the behaviour  4. Discriminative (learned) cue  *5. Prompts/cues* | 6. *Environmental changes (i.e. adding objects*  *to facilitate behaviour)* | Selected BCTs: 1, 5, 6  BCTs 1, 5 6: Provide automated notes detailing consultant advice on the test results (ideally provided on the end of the test results) and with cues for activation.  Non-selected BCTs: 2,3,4  BCT 2: Not possible in this context to restructure the social environment of GPs  BCT 3: not applicable for the target behaviour as aim is to promote effective testing among GPs where provided cues to support decision making is useful rather avoiding exposure to such support/cues.  BCT 4: Not within scope to offer any financial reward based on laboratory testing |
| **Beliefs about capabilities** | 1. Focus on past success  2. Verbal persuasion to boost self-efficacy | 3. Self-monitoring  4. Graded tasks, starting with easy tasks  5. Increasing skills: problem solving,  decision-making, goal-setting  6. Coping skills  7. Rehearsal of relevant skills  *8. Social process of encouragement, pressure*  *and support*  9. Feedback  10. Self-talk  11. Motivational interviewing | Selected BCTs: BCT 8 (mapped to professional role and identity – see description below)  Non selected BCTs: 1-7, 9-11  BCTs 1, 2, 6, 7, 9: Not feasible for this project due to variation in requesting patterns. For example GPs would require individually tailored verbal persuasion.  BCT 3: Immunogloblin tests often require advice from consultants/ lab scientists and the GP self-monitoring would not be feasible in this project.  BCTs 4, 5: not feasible due to likely lengthy time period and administration required to successfully implement.  BCTs 10, 11: Not applicable for immunoglobulin testing behaviour change. |
| **Beliefs about consequences** | 1. Vicarious reinforcement  2. Covert sensitisation  3. Covert conditioning  4. Emotional consequences  5. Threat  6. Pros and cons  7. Comparative imagining of future  outcomes | 8. Self-monitoring  *9. Persuasive communication (credible*  *source)*  *10. Information regarding*  *behaviour/outcome*  11. Feedback | Selected BCT: 9, 10  BCTs 9, 10: Clearly communicate situations where immunoglobulin testing is not beneficial (i.e. develop an algorithm of scenarios where tests should be performed, supported by consultant haematologists and GPs.  Non-selected BCTs: 1-8, 11  BCTs 1-7: Not applicable for immunoglobulin testing behaviours  BCT 8: Immunogloblin tests often require advice from consultants/ lab scientists and the GP self-monitoring would not be feasible in this project.  BCT 11: Not feasible for this project due to variation in requesting patterns. For example GPs would require individually tailored verbal persuasion. |
| **Professional role and identity** | No BCTs are linked to this domain | *1. Social process of encouragement, pressure,*  *support* | Selected BCTs: 1  BCT 1: Deliver a strategy developed by specialists and laboratory scientists’ in conjunction with GPs to provide the necessary feasible support/ guidelines on immunoglobulin testing procedures in primary care. |
| **Behavioural regulation** | 1. Self-monitoring of behaviour | *2. Goal/target specified behaviour or outcome*  3. Contract  4. Planning, implementation  *5. Prompts, triggers, cues*  6. Use of imagery | Selected BCTs: 2, 5  BCT 2, 5: Provide automated notes detailing consultant advice on the test results (ideally provided on the end of the test results) and with cues for activation.  Non- selected BCTs: 1, 3, 4, 6  BCT 1: Immunogloblin tests often require advice from consultants/ lab scientists and the GP self-monitoring would not be feasible in this project.  BCT 3: Contractual strategies not applicable for this laboratory testing behaviour change.  BCT 4, 6: Not applicable for this context of this project. |

*Selected BCTs are in italics
